# Exploring treatment-driven subclonal evolution of prognostic triple biomarkers: Dual gene fusions and chimeric RNA variants in novel subtypes of acute myeloid leukemia patients with KMT2A rearrangement

**DOI:** 10.1016/j.drup.2024.101199

**Published:** 2025-01-02

**Authors:** Yi Xu, Shengwen Calvin Li, Jeffrey Xiao, Qian Liu, Durga Cherukuri, Yan Liu, Saied Mirshahidi, Jane Xu, Xuelian Chen, Dadrastoussi Homa, Julian Olea, Kaijin Wu, Kevin R. Kelly, Fengzhu Sun, Ruihao Huang, Xiaoqi Wang, Qin Wen, Xi Zhang, Cristina M. Ghiuzeli, Esther Chong, Hisham Abdel-Azim, Mark E. Reeves, David J. Baylink, Huynh Cao, Jiang F. Zhong

**Affiliations:** a Loma Linda University Cancer Center, Loma Linda, CA 92354, United States; b Division of Hematology and Oncology, Department of Medicine, Loma Linda University, Loma Linda, CA 92354, United States; c Division of Regenerative Medicine, Department of Medicine, Loma Linda University, Loma Linda, CA 92354, United States; d Department of Neurology, University of California-Irvine School of Medicine, 200 S. Manchester Ave. Ste. 206, Orange, CA 92868, United States; e CHOC Children’s Research Institute, Children’s Hospital of Orange County (CHOC^®^), Rady Children’s Health, 1201 La Veta Ave, Orange, CA 92868-3874, United States; f Nevada Institute of Personalized Medicine, University of Nevada, Las Vegas, 4505 S Maryland Pkwy, Las Vegas, NV 89154, United States; g School of Life Sciences, College of Sciences, University of Nevada, 4505 S Maryland Pkwy, Las Vegas, NV 89154, United States; h Department of Pathology & Laboratory Medicine, Loma Linda University, Loma Linda, CA, United States; i Biospecimen Laboratory, Loma Linda University Cancer Center, Department of Medicine and Basic Sciences, Loma Linda University School of Medicine, Loma Linda, CA, United States; j Department of Basic Sciences, Loma Linda University, Loma Linda, CA 92354, United States; k Department of Medicine/Division of Hematology, University of Southern California, Los Angeles, CA, United States; l Quantitative and Computational Biology Department, University of Southern California, Los Angeles, CA, United States; m Medical Center of Hematology, Xinqiao Hospital of Army Medical University, Chongqing, China; n Division of Hematology, University of Washington School of Medicine, Seattle, WA, United States; o Division of Transplant and Cell Therapy/Hematological Malignancies, Departments of Pediatrics, Loma Linda University, Loma Linda, CA 92354, United States

**Keywords:** AML, Chromosomal Translocation, KMT2A, AFDN, Unknown Gene Fusion, RNA Variants, Relapse, Refractory

## Abstract

Chromosomal rearrangements (CR) initiate leukemogenesis in approximately 50 % of acute myeloid leukemia (AML) patients; however, limited targeted therapies exist due to a lack of accurate molecular and genetic biomarkers of refractory mechanisms during treatment. Here, we investigated the pathological landscape of treatment resistance and relapse in 16 CR-AML patients by monitoring cytogenetic, RNAseq, and genome-wide changes among newly diagnosed, refractory, and relapsed AML. First, in FISH-diagnosed KMT2A (MLL gene, 11q23)/AFDN (AF6, 6q27)-rearrangement, RNA-sequencing identified an unknown CCDC32 (15q15.1)/CBX3 (7p15.2) gene fusion in both newly diagnosed and relapsed samples, which is previously unknown in KMT2A/AFDN-rearranged AML patients. Second, the unreported CCDC32/CBX3 gene fusion significantly affected the expression of wild-type genes of both CCDC32 (essential for embryonic development) and CBX3 (an oncogene for solid tumors) during the relapse, as demonstrated by Quantitative PCR analyses. Third, we further confirmed the existence of triple biomarkers - KMT2A/AFDN (AF6, 6q27) rearrangement, the unknown CCDC32 (15q15.1)/CBX3 (7p15.2) gene fusion and chimeric RNA variants (treatment-resistant leukemic blasts harboring distinct breakpoints) in a 21-year-old male patient of rapid relapsed/refractory AML. Most intriguingly, in this work regarding 16 patients, patients 7 and 20 initially showed the KMT2A/AFDN gene fusion; upon relapse, patient 20 did not show this fusion. On the other hand, patient 7 retained the KMT2A/AFDN fusion at diagnosis and during the relapse, only identified by PCR and Sanger’s Sequencing, not by cytogenetics. Interestingly, the chimeric CCDC32/CBX3 gene fusion persisted in the 21-year-old male patient over the diagnostic and relapse phases. Most intriguingly, the overexpression of CCDC32/CBX3 fusion gene in AML patient-specific MV4–11 cells confirms the functional validation, providing experimental evidence of the biological impact of the CCDC32/CBX3 fusion on AML pathogenesis and treatment resistance by promoting cell cycle progression, a mechanism through which AML evolves to become treatment-resistant. All these might exhort differential resistance to treatment. Thus, we found that prognostic and predictive triple biomarkers - KRAS mutated, dual fusions (KMT2A/AFDN, CCDC32/CBX3), and chimeric variants - might evolve with a potential oncogenic role of subclonal evolution for poor clinical outcomes.

## Introduction

Chromosomal rearrangements (CR) serve as a pivotal indicator in the pathology of acute myeloid leukemia (AML), the predominant form of leukemia in adults (Dohner et al., 2015; Kumar, 2011). Approximately half of all AML cases exhibit chromosomal abnormalities that give rise to fusion proteins, significantly contributing to the disease’s ontogenesis and its recurrence or refractory nature. Notably, rearrangements involving the gene lysine methyltransferase 2a (KMT2A) at 11q23 rank among the most frequent chromosomal abnormalities in AML, often leading to treatment resistance and relapse (Bill et al., 2020; Issa et al., 2021). The KMT2A gene has over 80 known fusion partners, one of which, AFDN (AF6, 6q27), activates the oncogenic RAS pathway, further promoting AML progression (Winters and Bernt, 2017). The translocation t(6;11)(q27;q23), resulting in a KMT2A/AFDN fusion gene, is linked to poor clinical outcomes (Kantarjian, 2016; Winters and Bernt, 2017), thus emphasizing the urgent need for new therapeutic approaches for patients with KMT2A-rearranged AML, which led our efforts of searching for alternatives biomarkers.

Unknown gene fusions have recently emerged from pediatric leukemia studies that have been reported in pediatric leukemia patients (Berg et al., 2021; Berg et al., 2022; Hoffmeister et al., 2021). Current clinical guidelines require chromosome analysis, FISH testing, and/or molecular testing of known genetic abnormalities in newly diagnosed AML patients as part of the treatment strategy (Thol, 2022; Wheeler et al., 2018). However, these routine clinical tests fail to detect novel unknown translocations and mutations, leading to possibly missed therapeutic options for targeting AML biomarkers (Arindrarto et al., 2021). The advent of next-generation Sequencing (NGS), mainly through transcriptome sequencing (RNA-seq), has catalyzed the discovery of unknown gene fusions not identifiable through conventional chromosomal analyses(Li et al., 2014). Likewise, whole-genome sequencing (WGS) has demonstrated enhanced diagnostic accuracy over traditional cytogenetic analyses in discerning genomic level abnormalities in AML and myelodysplastic syndromes (MDS).

This study posits that, akin to pediatric cases, adult AML patients harbor unknown gene fusions that might play a crucial role in treatment resistance and disease recurrence. Such gene fusions might go through subclonal evolution with drug resistance and relapse as we previously hypothesized of subclonal cross-talk signaling transduction (Li et al., 2012) using our single-cell transcriptome technology(Lee and Li, 2020). Here, by integrating RNA-seq with PCR/Sanger DNA-sequencing beyond standard chromosome and FISH analyses, we aimed to map out the fusion gene landscape in newly diagnosed AML patients with KMT2A rearrangement and throughout their treatment courses. Our findings suggest that beyond persistent oncofusion proteins, unknown fusion genes and chimeric RNA variants could serve as alternative survival mechanisms in relapsed or refractory AML settings.

## Materials and methods

The qPCR and PCR primers list is in the supplementary data (Supplementary Table 1). Replicates (n = 3) were performed for all experiments.

### Human specimens

Bone marrow samples of seven AML patients (Table 1) at different time points (at the time of initial diagnosis defined as newly diagnosed and after treatment or relapse) were obtained from the Loma Linda University Cancer Center Biospecimen Laboratory (LLUCCBL). All patients signed an informed consent. This study was approved by the Institutional Review Board of Loma Linda University and conducted according to the Declaration of Helsinki.

### Chromosome analysis (Karyotyping)

Chromosome analysis of the bone marrow samples was performed according to the standard clinical laboratory protocols. All GTG-banded slides were scanned, and a total of twenty metaphase cells were analyzed for each case using the ASI imaging system (Applied Spectral Imaging, Carlsbad, CA).

### Fluorescent in situ hybridization (FISH) analysis

The *KMT2A* (also known as *MLL*) gene rearrangement testing on the bone marrow samples was performed using the KMT2A break-apart FISH probe (OGT Inc., New York, NY). A KMT2A-AFDN dual color, dual fusion FISH probe was used to detect the KMT2A-AFDN gene fusion. A total of 200–500 interphase nuclei were analyzed.

### RNA-sequencing (RNA-seq)

Human bone marrow (BM) mononuclear cells were isolated from AML patients 7 and 20 by Ficoll-Paque^™^ PLUS (GE Healthcare). High-quality total RNA was isolated using the Qiagen RNeasy Mini Kit (Qiagen). Sequencing libraries were prepared by TruSeq Stranded mRNA Library Prep Kit (Illumina) and then sent to Novogene for 150 bp pairend, 30 million reads sequencing (Novogene Corporation Inc., CA, USA). The Sequencing generated ~27.7 million pair-end (PE) short reads for patient 7 and ~22.1 million PE short reads for patient 20, and the read length was 150 bp. We separately aligned the short reads against the human reference genome assembly GRCh38/hg38 for each patient. During alignment, we used STAR v2.7.10a with the gene annotation GENCODE v41. After alignment, we used Arriba v2.3.0 to detect gene fusions. We also filtered gene fusions where two genes were from identical chromosomes and adjacent to each other according to the gene definition in GENCODE. After filtering, multiple candidate gene fusions were acquired for further analysis for patients 7 and 20. The raw data sets were deposited onto the database of the NCBI (The National Center for Biotechnology Information) of the NIH (the National Institutes of Health) with the bioproject number PRJNA1201248.

### RNA isolation and quantitative real-time polymerase chain reaction (qPCR) analysis

As previously described, BM cells from patient specimens were collected for RNA isolation and qPCR analysis (Xu et al., 2022b). Total RNA was isolated using the RNeasy Mini Kit (Qiagen) according to the manufacturer’s instructions. First-strand complementary DNA (cDNA) was synthesized by reverse transcriptase-polymerase chain reaction (RT-PCR) using the SuperScript III Reverse Transcriptase (Invitrogen, Waltham, MA, USA). The qPCR was performed and analyzed with an Applied Biosystems 7900HT Real-Time PCR machine. The primer sequences used to amplify different target regions are presented in Supplementary Table 1. PCR conditions were 10 min at 95^◦^C followed by 40 cycles of 10 s at 95^◦^C and 15 s at 60^◦^C. A gene’s relative expression level was determined using the ΔΔCt method and normalized to β-actin.

### Polymerase chain reaction (PCR) and Sanger DNA-sequencing

The breakpoints of t(6;11) chromosome translocation at the DNA level were detected through PCR followed by Sanger DNA sequencing. Genomic DNA (gDNA) was isolated using the QIAamp DNA Mini Kit (Qiagen) according to the manufacturer’s instructions. PCR was performed with an Applied Biosystems 2720 Thermal Cycler (Fisher Scientific) to amplify products using gDNA and cDNA as previously described (Xu et al., 2008). Primers were designed to span the fusion genes’ putative breakpoints using Primer3 web software (Untergasser et al., 2012). The sequences and locations of the primers are available in Fig. 2D (**KMT2A::AFDN fusion)**, Fig. 3C **(CCDC32::CBX3 fusion)**, and Supplementary Table 1. After gel electrophoresis, PCR products were purified from the agarose gel with the QIAquick Gel Extraction Kit (Qiagen), according to the manufacturer’s instructions. PCR amplicons were Sanger DNA-sequenced by MCLAB (Molecular Cloning Laboratories, South San Francisco, CA, USA). Sequence alignments were analyzed using BLAST (NCBI) and BLAT (UCSC Genome Browser).

### Functional validation of the CCDC32/CBX fusion gene: generation of a new transgenic cell line overexpressing the CCDC32/CBX fusion gene in Vitro

The custom-built lentiviral construct of the CCDC32/CBX3 fusion gene (Fig. 9) was obtained from GeneCopoeia with catalog # CS-GS2239L-Lv225–01. The lentiviral expression construct contains CCDC32/CBX3 fusion gene and eGFP reporter, with promoters EF1a and IRES2, respectively. Lentiviruses were prepared as previously described (Xu et al., 2022b). Briefly, HEK-293T cells were cultured in complete Dulbecco’s modified Eagle’s medium (DMEM, Gibco, Waltham, MA, USA) containing 10 % FBS and 100 U/mL penicillin/streptomycin. When the cells were 70–80 % confluent, the culture media was replenished, and a transfection solution containing envelope, packaging, and transfer plasmid of the CCDC32/CBX3 fusion gene was added dropwise to the cells. Afterward, the cells were cultured at 37 ^◦^C and 5 % CO_2_ for 48 h, filtered through a 0.45 μm filter, and centrifuged at 4800 g at 4 ^◦^C for 24 h. The virus pellet was reconstituted in PBS containing 5 % glycerol and titrated.

A GFP empty vector (GeneCopoeia catalog#: EX-NEG-Lv225) was used as the vector control. The MV4–11 cells were transduced with the lentivirus of the CCDC32/CBX3 fusion gene at a multiplicity of infection (MOI) of 5. Twenty-four hours later, the virus was removed, and the culture medium was replenished. The new cell line of CCDC32/CBX3-MV4–11 was further experimentally validated via microscopy, gene expression, and flow cytometry assays.

### Imaging acquisition

Immunocytochemistry and imaging acquisition were performed as previously described (Xu et al., 2022a). Phase-bright and fluorescent images were taken using an Olympus 1 × 71 inverted microscope and were processed using an Olympus cellSens Dimension 1.15 Imaging Software.

### Flow cytometry (FC)

Cells were harvested and examined for the expression of viability dye, cell surface biomarkers, and intracellular proteins by multichromatic FC, as previously described (Cao et al., 2020). The viability dye used in this study is Fixable Viability Dye eFluor^™^ 780 (eBioscience Cat#: 65–0865–14). Briefly, for FC staining, after the staining of viability dye, about 1 × 10^4^ ~ 10^6^ cells in 100 μl FC buffer (PBS containing 1 % FBS and 0.05 % sodium azide) were stained with desired cell surface proteins at 4 ^◦^C for 30 min. The surface-stained cells were then fixed, permeabilized using the appropriate reagents (BD Pharmingen Cytofix/Cytoperm buffer), and further stained with Ki67 (Biolegend catalog#: 350504) at 4 ^◦^C for 2 h. Concentrations of antibodies and dyes were applied according to the manufacturers’ recommendations. Finally, the stained cells were detected on the BD FACSAria II. FC Data was analyzed using the FlowJo software (Tree Star Inc., Ashland, OR).

Sequences of qPCR primers (OriGene)

Human CDK1

Forward Sequence GGAAACCAGGAAGCCTAGCATC

Reverse Sequence GGATGATTCAGTGCCATTTTGCC

Human Cyclin B1 (CCNB1)

Forward Sequence GACCTGTGTCAGGCTTTCTCTG

Reverse Sequence GGTATTTTGGTCTGACTGCTTGC

### Statistical analysis

Statistical analyses were performed with GraphPad software (Prism version 5, San Diego, CA, USA). The quantitative comparison between the two groups was analyzed using an unpaired *t*-test. All values were presented as mean ± SEM (Standard Error of the Mean). Results were considered statistically significant when the p-value was < 0.05.

(the novel AML fusion gene was detected n = 2 out of 16 patients screened)

## Results

**Fig. 1.**
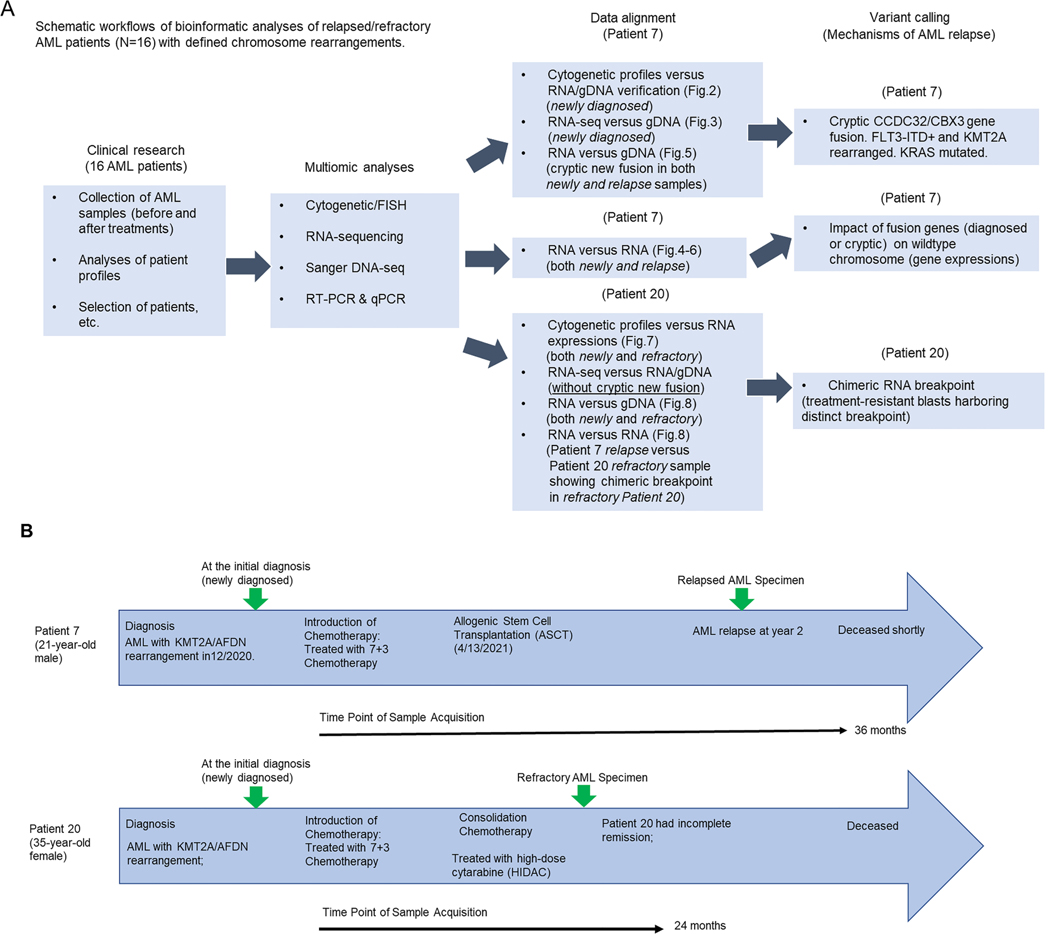
Experimental design of this study and a flow chart of treatment schemes. A) Schematic illustration of experimental design and bioinformatic analyses of this study; All AML samples were acquired from both stages: before treatment (newly diagnosed) and after treatment (either relapse or refractory) and were experimentally investigated at both genomic (gDNA) and RNA (cDNA) levels. B) Clinical courses of patients 7 and 20 with time points of specimen acquirement and treatment timelines. [ITD stands for “internal tandem duplication,” referring to a mutation where a segment of DNA is duplicated within the gene. In the context of FLT3 (Fms-like tyrosine kinase 3), FLT3-ITD mutation involves the insertion of tandem repeats of nucleotides in the juxtamembrane domain of the FLT3 gene. This mutation is commonly found in acute myeloid leukemia (AML) and is associated with poor prognosis.] (The “7 +3 chemotherapy” is a treatment regimen used for certain types of leukemia, particularly acute myeloid leukemia (AML). It consists of cytarabine (also known as Ara-C) and an anthracycline antibiotic, typically daunorubicin or idarubicin. The “7 + 3” refers to a schedule where cytarabine is given for 7 days continuously, and the anthracycline is given for 3 days. This regimen is commonly used as induction chemotherapy to induce remission in patients with AML). (n = 2 patients).

We performed molecular and cytogenetic analyses of bone marrow aspirates from 16 different adult *de novo* AML patients to identify candidates for the study of KMT2A-rearranged gene fusions as a primary screening (Table 1). Two patients, 7 and 20, were identified to possess the chromosomal translocation t(6;11) (q27;q23) at the initial diagnosis of AML, which was predicted to lead to KMT2A/AFDN gene fusion (Fig. 1A). Both patients deceased shortly after different treatment regimens (Fig. 1B). We hypothesized that an unknown mechanism by which patient 7 underpinned his rapid AML progression, and we retrospectively integrated multiple lines of analyses, including cytogenetics, RNA sequencing, genomic Sequencing, and bioinformatics on the patient’s bone marrow samples collected at the time of diagnosis as well as relapse (Fig. 1A).

### Clinical courses

Patient 7 was a 21-year-old male presenting with a white blood cell count (WBC) of 42,000 cells/μl and 76 % blasts (immature or undifferentiated cells found in the blood or bone marrow) that was consistent with a diagnosis of acute leukemia, such as acute myeloid leukemia (AML), manifested with t(6;11)/11q23/MLL rearrangement, Hypercellular marrow for age (~90 % cellularity) with foci of residual trilineage hematopoiesis bone marrow, aspirate/imprints/buffy-coat smears, clot sections, and trephine core biopsy, left posterior iliac crest, indicating his AML FISH: KMT2A (MLL) 11q23 rearrangement (Chromosome: 46, XY,t(6,11)(q27;q23)[11]/46, XY[9]), biomarker KRAS mutated (unfavorable risk), MDS FISH: Normal at diagnostics of December 7, 2020. The status post chemotherapy regimen and subsequently minimal residual disease-negative. status post-HIDAC (high-dose cytarabine) chemotherapy and status post minimal residual disease-negative with (brother) allogeneic hematopoietic stem cell transplantation. Patient 7 initially went into complete remission after standard chemotherapy, followed by allogeneic stem cell transplantation. However, the disease relapsed two years later, and he expired shortly thereafter.

**Fig. 2.**
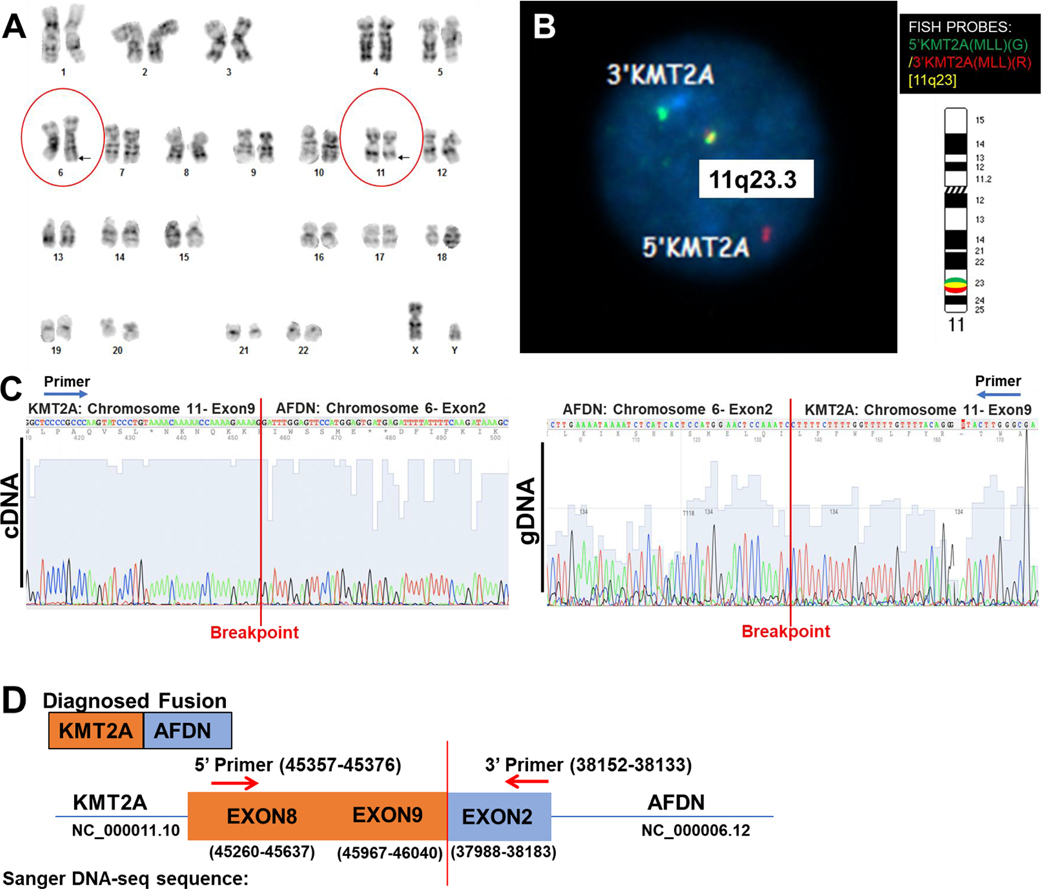
Detection of KMT2A/AFDN chromosome translocation in patient 7. A) Chromosomal karyotype image showing a translocation between chromosomes 6 and 11 (black arrows in red circles). B) Fluorescent in-situ hybridization (FISH) image showing an abnormal interphase in patient 7 with a KMT2A gene rearrangement. The KMT2A 5′ (green)/KMT2A 3′ (red) break-apart FISH probe was hybridized to the chromosome 11q23.3 region. Please note the presence of one fusion signal, one green, and one red signal. The separation of green and red signals suggests KMT2A gene rearrangement. C) Chromatogram of Sanger DNA sequencing showing the cDNA (left panel) and gDNA (right panel) of newly diagnosed patient 7 with breakpoints flanked by the KMT2A (chromosome 11, exon 9) and AFDN (Chromosome 6, exon 2) genes.D) Graphic illustration of KMT2A/AFDN fusion and DNA sequence of breakpoint and primers.

Specifically, his chromosome analysis of the bone marrow sample at the time of initial diagnosis revealed an apparently balanced translocation between the long arms of chromosomes 6 and 11, t(6;11)(q27; q23) as the sole abnormality (Fig. 2A). Fluorescent in situ hybridization (FISH) was subsequently performed to examine KMT2A-rearrangement in interphase nuclei. Instead of two colocalized red and green (yellow) signals (indicating intact KMT2A gene), the KMT2A break-apart FISH probe showed the presence of one colocalized yellow signal and individual green and red signal patterns, suggesting rearrangement of the KMT2A gene (Fig. 2B). To experimentally validate whether the t(6;11) translocation identified in this sample led to the KMT2A::AFDN gene fusion, PCR with specifically designed primers was performed, followed by Sanger DNA-sequencing on patient 7’s cDNA (complementary DNA) and gDNA (genomic DNA) obtained at diagnosis (Fig. 2C & D). Analysis of these results showed that patient 7 had a chimeric chromosome with a breakpoint flanked by exon 9 of KMT2A and exon 2 of the AFDN gene (KMT2A exon 9::AFDN exon 2) (Fig. 2C & D). These data confirm that the translocation of the t(6;11) resulted in KMT2A/AFDN gene fusion in this bone marrow sample.

### Identification of CCDC32/CBX3 fusion previously unknown in the AML bone marrow sample collected at diagnosis

**Fig. 3.**
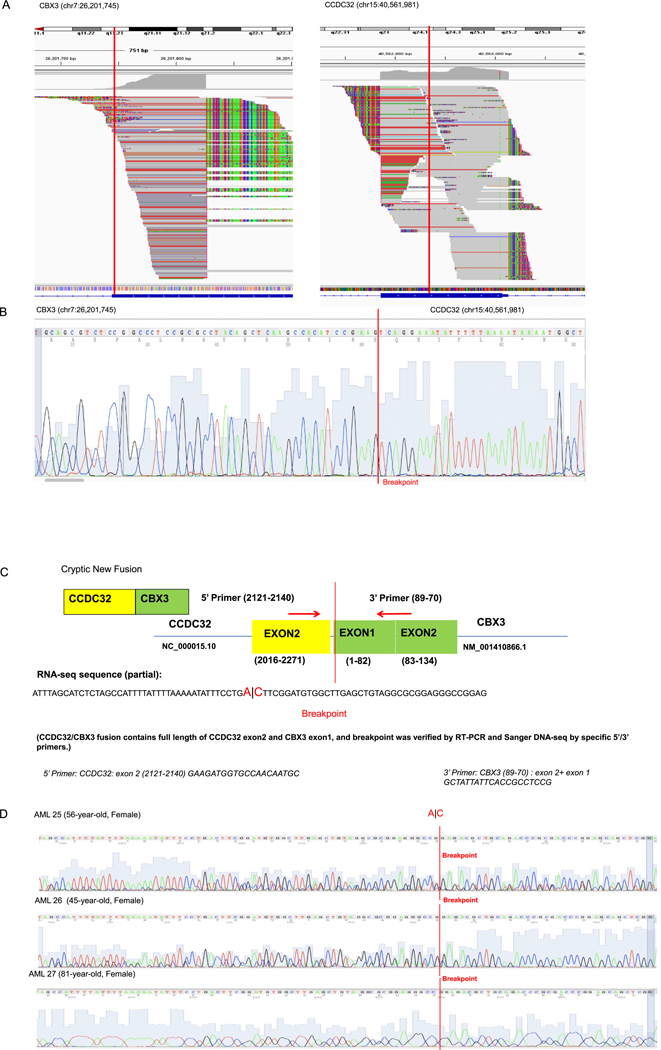
Identify an unknown fusion gene (CCDC32/CBX3) in patient 7 at diagnosis. A) RNA sequencing analysis shows a breakpoint (vertical red line) in exon 1 of CBX3 (chr7:26,201,745) (left panel) and a breakpoint in exon 2 of CCDC32 (chr15:40,562,981) (right panel).B) Chromatogram of Sanger DNA sequencing showing a breakpoint flanked by CBX3 (chr7:26,201,745) and CCDC32 (chr15:40,561,981) at the time of diagnosis.C) Graphic illustration of CCDC32/CBX3 fusion and DNA sequence of breakpoint and primers. D) Chromatogram of Sanger DNA sequencing confirming that AML patients 25, 26, 27 had the crypt fusion gene CCDC32/CBX3 with a breakpoint flanked by CBX3 (chr7:26,201,745) and CCDC32 (chr15:40,561,981) at the time of diagnosis.

To explore our hypothesis that, like pediatric leukemia, unknown gene fusions might be present in KMT2A-rearranged adult AML, we performed transcriptome-wide surveillance for fusion genes using RNA sequencing (RNA-seq) (Heyer et al., 2019). The RNA-seq analysis detected a CCDC32/CBX3 gene fusion with breakpoints located in the exon 1 of CBX3 (chr7:26,201,745) and exon 2 of CCDC32 (chr 15:40, 562,869) (Fig. 3A), suggesting the dual gene fusion in patient 7. PCR with specifically designed primers and subsequent Sanger DNA-sequencing confirmed the presence of CBX3 (7p15.2)::CCDC32 (15q15.1) gene fusion in both cDNA (Fig. 3B & C) and gDNA (Fig. 5A). The chromosome analysis showed no visible structural rearrangement between chromosomes 7 and 15, consistent with an unknown nature of the CBX3/CCDC32 gene fusion in patient 7. Next, to examine whether other AML patients also possess this novel crypt CBX3/CCDC32 gene fusion, we acquired additional 9 AML patient samples (Patient 23–31). Indeed, we found the presence of CBX3 (7p15.2)::CCDC32 (15q15.1) gene fusion in the cDNA of an additional three female patients. The PCR screening was performed using specifically designed primers and patient 7 samples as the positive control. The PCR products were further confirmed by subsequent Sanger DNA-sequencing, showing the same breakpoint of AML patients 25, 26, and 27 as patient 7 (Fig. 3D). Thus, we identified a novel CBX3 (7p15.2)::CCDC32 (15q15.1) gene fusion in AML patients, which has a frequency of 25 % (4 +/16 patients) among our screened AML cases.

### No KMT2A/AFDN gene fusion was detected in the bone marrow sample during relapse via cytogenetic analysis, but it was found through PCR and Sanger DNA sequencing of RNA samples

**Fig. 4.**
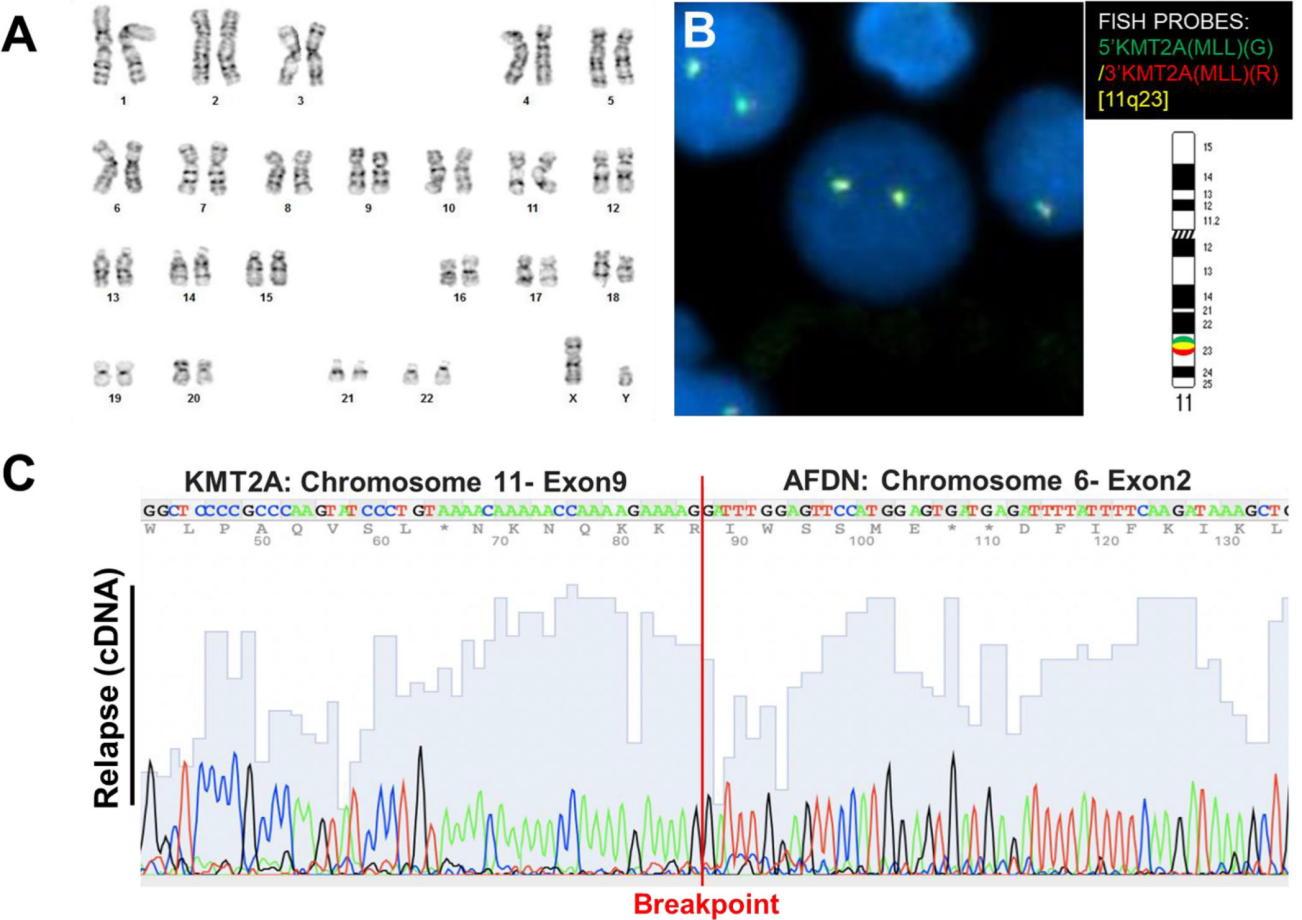
Cytogenetic analyses show the absence of KMT2A rearrangement in the relapsed bone marrow sample of patient 7.A) Chromosome analysis shows no observable t(6;11) translocation in the relapsed bone marrow sample.B) Fluorescent in-situ hybridization image with KMT2A 5′ (green)/KMT2A 3′ (red) break-apart FISH probes (11q23.3 region) showing two colocalized red and green (yellow) signals, suggesting the absence of KMT2A gene rearrangement. C) Chromatogram of Sanger DNA-seq showing the observed breakpoint in the cDNA reversely transcribed from patient 7’s RNA isolated from the bone marrow sample at relapse.

Patient 7 experienced disease relapse approximately two years after the initial diagnosis. Chromosome analysis and FISH testing were performed on the relapsed bone marrow sample. Our chromosomal analysis revealed a normal karyotype with no observable t(6;11) translocation (Fig. 4A). Additionally, FISH analysis using the KMT2A breakapart probe showed no evidence of KMT2A gene rearrangement (Fig. 4B). However, PCR and Sanger DNA sequencing of the RNA samples during the relapse confirmed the presence of the diagnosed KMT2A/AFDN gene fusion, with the same breakpoint flanked by KMT2A exon 9::AFDN exon 2 (Fig. 4C). This result suggests that PCR is more sensitive in detecting fusion genes at the RNA level than routine cytogenetic approaches for analyzing genomic and structural changes.

### Detection of CCDC32/CBX3 gene fusion at both cDNA and gDNA

**Fig. 5.**
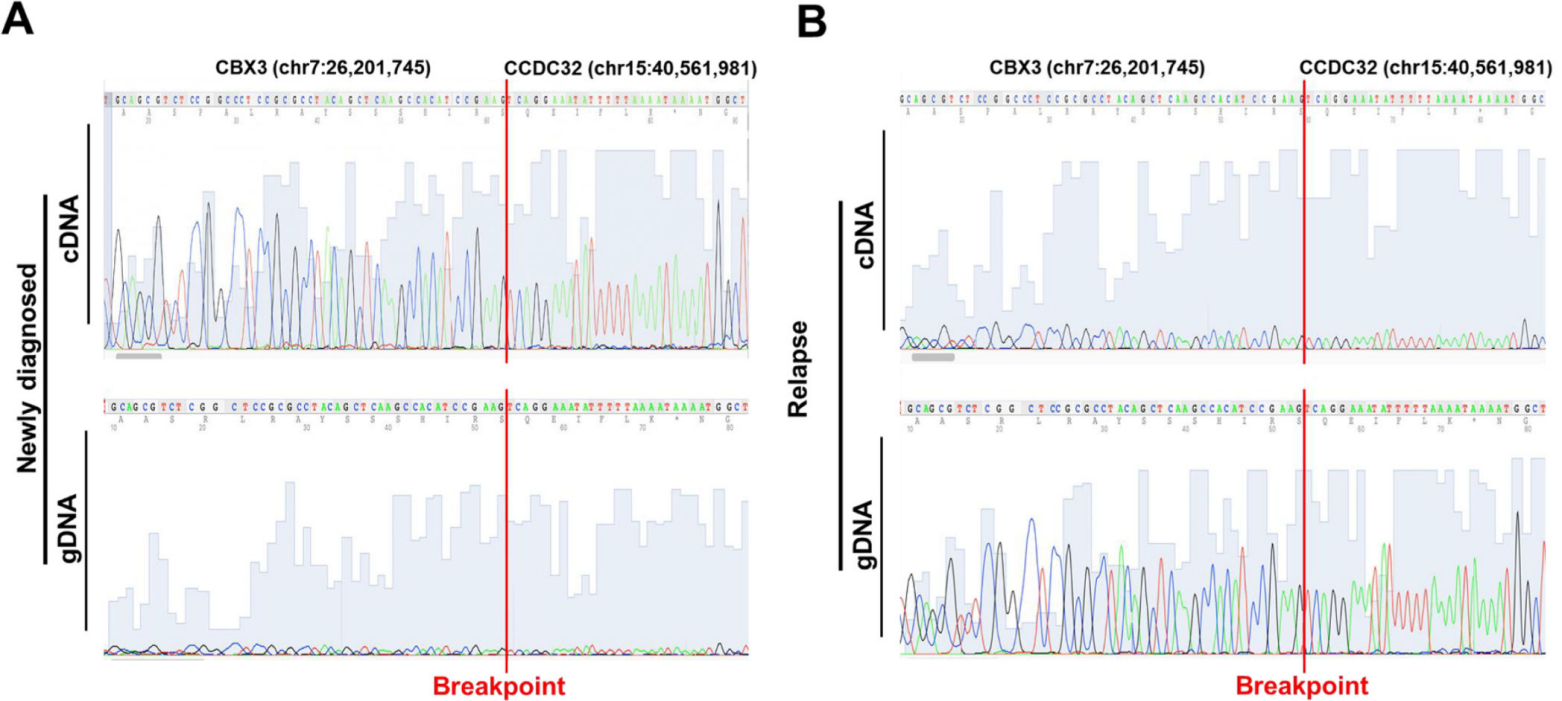
Detection of the unknown CCDC32/CBX3 gene fusion in cDNA and gDNA extracted from the bone marrow samples collected at diagnosis and at the time of relapse of patient 7. A) Chromatogram of Sanger DNA-seq showing the breakpoint (vertical red line) of CCDC32/CBX3 fusion gene in the cDNA (top panel) and gDNA (bottom panel) at the time of diagnosis. B) Chromatogram of Sanger DNA-seq showing the breakpoint (vertical red line) of CCDC32/CBX3 fusion gene in the cDNA (top panel) and gDNA (bottom panel) at the time of relapse.

After detecting the unknown CCDC32/CBX3 fusion gene in the bone marrow sample of Patient 7 at diagnosis, we examined the bone marrow sample collected at the time of relapse for the presence of the CCDC32/CBX3 gene fusion. Sanger sequencing experiments confirmed the presence of the CCDC32/CBX3 fusion gene in both cDNA and gDNA of the relapsed bone marrow sample, with breakpoints identical to those observed in the bone marrow sample at diagnosis (Fig. 5A and B). These findings suggest that the CCDC32/CBX3 gene fusion persisted throughout the disease and was unaffected by treatment. Dynamic changes in expression of KMT2A/AFDN and CCDC32/CBX3 gene fusions and their wild-type counterparts at diagnosis and relapse

**Fig. 6.**
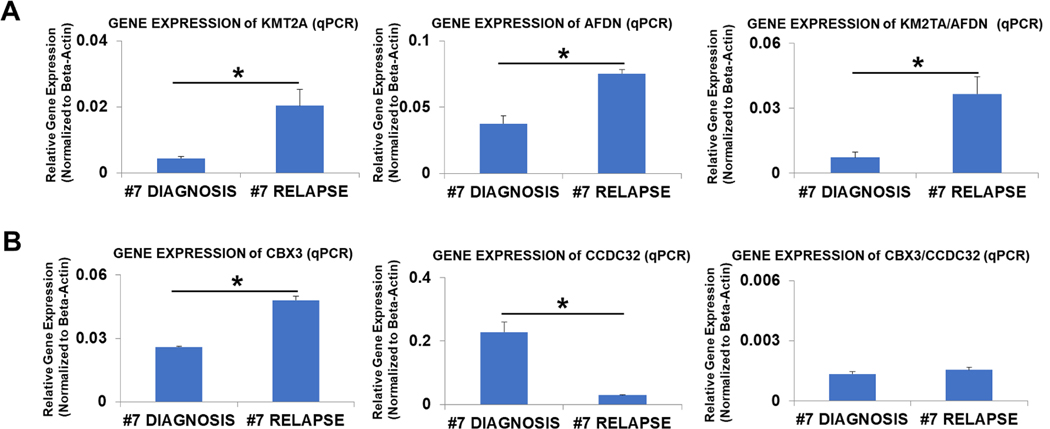
Dynamic expression changes of chimeric KMT2A/AFDN and CCDC32/CBX3 wild-type genes in the newly diagnosed (at diagnosis) and the relapsed samples of patient 7.A) qPCR analysis of the gene expressions of KMT2A, AFDN, and KMT2A/AFDN fusion in newly diagnosed versus after relapse for patient 7; Data of mRNA expressions (normalized to β-actin) of KMT2A gene (left panel), AFDN gene (middle panel), and KMT2A/AFDN fusion gene (right panel). B) qPCR analysis of the gene expressions of CBX3, CCDC32, and CCDC32/CBX3 fusion in newly diagnosed versus after relapse for patient 7; Data of mRNA expressions (normalized to β-actin) of CBX3 gene (left panel), CCDC32 gene (middle panel), and CCDC32/CBX3 fusion gene (right panel). Wherever applicable, data are means ± SEM and were analyzed by student “t” test. *P < 0.05, n = 3.

To further characterize whether the treatment had any effect on the expression levels of KMT2A/AFDN and CCDC32/CBX3 gene fusions, we employed quantitative PCR (qPCR) to examine their expression levels in the bone marrow samples collected at diagnosis and relapse using the expression of their wild-type counterparts as controls. We observed a substantial rise of 4.7-fold in wild-type KMT2A (Fig. 6A, left panel) and a 2-fold increase in AFDN (Fig. 6A, middle panel) compared to the initial diagnosis and relapse, respectively. Nevertheless, qPCR results unveiled a striking 5-fold surge in the expression of the chimeric KMT2A/AFDN fusion gene during the relapse (Fig. 6A, right panel).

When we looked into how the chimeric CCDC32/CBX3 fusion altered during the relapse, we uncovered a distinct trend when examining the changes in CCDC32/CBX3 fusion during the relapse. Notably, wild-type CBX3 exhibited a notable 1.9-fold increase (Fig. 6B, left panel). In contrast, wild-type CCDC32 showed a marked decrease of 7.4-fold during the relapse compared to the initial diagnosis (Fig. 6B, middle panel). Interestingly, no significant difference was observed in the CCDC32/CBX3 gene fusion expression between the relapse and the initial diagnosis (Fig. 6B, right panel).

Upon retrospective analysis, we discovered a hidden CCDC32 (15q15.1)/CBX3 (7p15.2) gene fusion in patient 7, which was initially diagnosed with a KMT2A/AFDN rearrangement. This fusion was found in both initial and relapsed cases. Additionally, combining qPCR, Sanger DNA sequencing, RT-PCR, and RNA-seq assays improved the precision of diagnostic profiling and complemented standard cytogenetic analysis. We wished to validate the profiling of patient 7 in a different patient.

### The clinical trajectory varies among individual patients

**Fig. 7.**
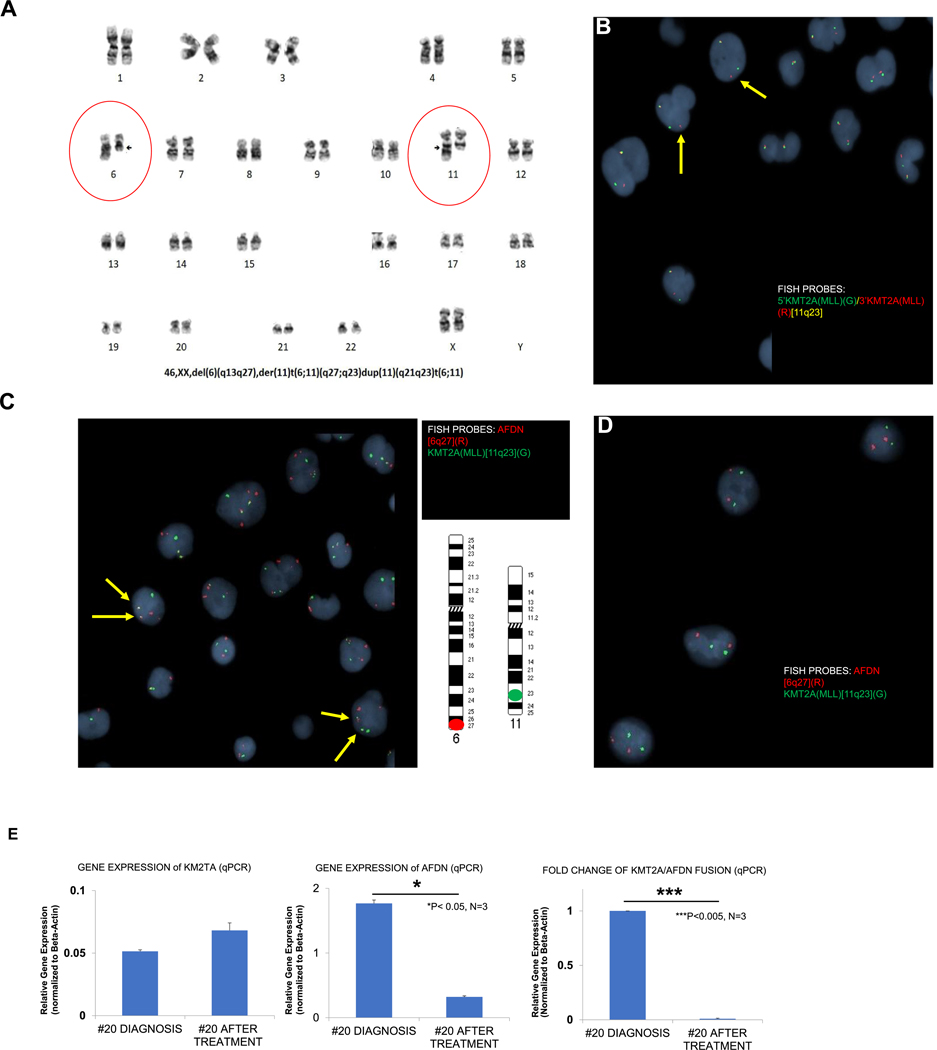
Clinical Profile and expression changes of wild-type (KMT2A or AFDN) and fusion (KMT2A/AFDN) genes in newly diagnosed (diagnosis) and refractory (after treatment) patient 20.A) Chromosomal karyotype image showing a translocation between chromosomes 6 and 11 in patient 20 (black arrows in red circles). B) Fluorescent in-situ hybridization (FISH) image showing an abnormal interphase in patient 20 and the KMT2A gene rearrangement. The KMT2A 5′ (green)/KMT2A 3′ (red) breakapart FISH probes hybridize to the chromosome 11q23 region. A chimera chromosome possesses either the 5′ KMT2A (red) only or the 3′KMT2A (green) only, indicated by yellow arrows. C) FISH image of patient 20 showing a fusion profile (yellow arrows) of AFDN and KMT2A (MLL) in the diagnostic specimen. Chimeric KMT2A/AFDN is displayed by the yellow color. D) FISH image of patient 20 showing a non-fusion profile of AFDN and KMT2A (MLL) in the after-treatment follow-up specimen. The AFDN (red) FISH probes hybridize to the chromosome 6q27 region, while the KMT2A (green) FISH probes hybridize to the chromosome 11q23 region. Chimeric KMT2A/AFDN is displayed by the yellow color. E) qPCR analysis of the gene expressions of KMT2A, AFDN, and KMT2A/AFDN fusion before and after treatment for patient 20. Data of mRNA expressions (normalized to β-actin) of the KMT2A gene (left panel), AFDN gene (middle panel), and KMT2A/AFDN fusion gene (right panel). Where applicable, data are means ± SEM and were analyzed by student “t” test. *P < 0.05, * **P < 0.005, n = 3.

Clinical courses can differ significantly between patients. To validate the profiling of Patient 7, we examined another AML patient with a KMT2A/AFDN rearrangement. Patient 20, a 35-year-old woman diagnosed with AML with monocytic differentiation, presented with a WBC count of 17,800 cells/μL and 80 % blasts, confirmed by both flow cytometry and immunohistochemistry. She underwent (7 +3) induction chemotherapy followed by high-dose cytarabine (HIDAC) for consolidation. Chromosome analysis of her bone marrow sample revealed an apparently unbalanced translocation between the long arms of chromosomes 6 and 11, t(6;11)(q27;q23) (Fig. 7A).

Subsequently, FISH testing was conducted using a KMT2A break-apart probe to investigate the presence of KMT2A-rearrangement in interphase nuclei. The KMT2A break-apart FISH probe revealed the existence of one colocalized yellow signal along with separate green and red signal patterns, indicating the occurrence of a KMT2A-rearrangement (arrows, Fig. 7B). Subsequently, the FISH analysis using a KMT2A-AFDN dual color, dual fusion probe revealed an atypical FISH signal pattern characterized by the presence of two fusions - one green and one red signal. This pattern indicates the KMT2A/AFDN gene fusion in the patient’s recently diagnosed specimen, as depicted in Fig. 7C. After treatment, a bone marrow sample was subjected to FISH testing using a KMT2A-AFDN dual color, dual fusion-gene probe. The results indicated a normal signal pattern, with two green and two red signals indicating the absence of KMT2A/AFDN gene fusion (Fig. 7D). This result suggests that drug-treatment-driven events have led to a subclonal evolution, a concept we previously hypothesized (Li et al., 2012).

### Treatment-driven gene expression changes of wild-type and fusion genes

Considering the notable changes observed in the expression patterns of wild-type genes and chimeric gene fusions during the relapse versus the initial diagnosis in Patient 7, we proceeded to investigate whether similar changes occurred in the gene expression of wild-type genes (KMT2A or AFDN) and the KMT2A/AFDN gene fusion following treatment in Patient 20.

Subsequent qPCR analysis revealed that the expression of the KMT2A wild-type gene increased by 1.3-fold after treatment compared to before treatment (Fig. 7E, left panel). In contrast, the AFDN wild-type gene expression significantly decreased by 5.5-fold after treatment (Fig. 7E, middle panel). Remarkably, the gene expression of the KMT2A/AFDN fusion drastically decreased by 103-fold after treatment (Fig. 7E, right panel). These findings suggest a substantial reduction in KMT2A/AFDN fusion mRNA following treatment.

### Chimeric variations of the KMT2A/AFDN gene fusion between different patients

**Fig. 8.**
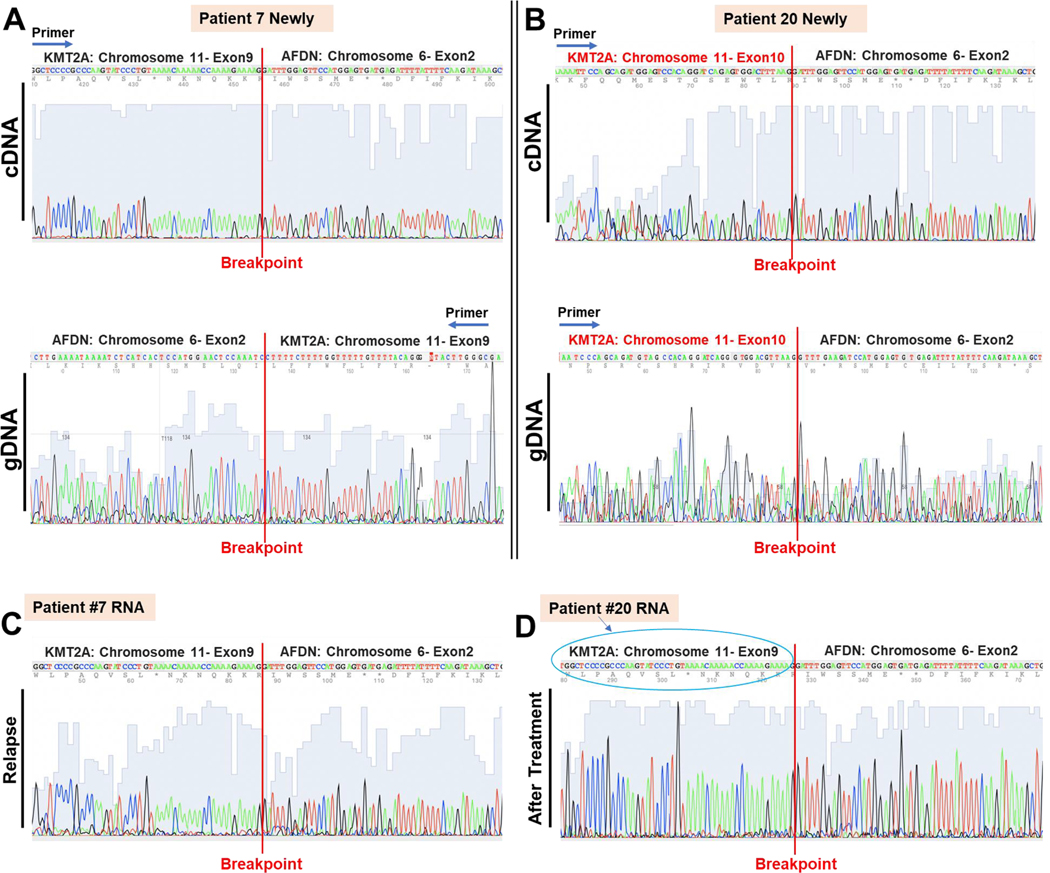
Comparison of chimera KMT2A/AFDN gene breakpoints in newly diagnosed and refractory patients 7 and 20, revealing the chimeric RNA variants in refractory patient 20.A) Chromatogram of Sanger DNA-seq showing the cDNA and gDNA of newly diagnosed patient 7’s breakpoint flanked by KMT2A (chromosome 11, exon 9) and AFDN (Chromosome 6, exon 2).B) Chromatogram of Sanger DNA-seq showing the cDNA and gDNA of newly diagnosed patient 20’s breakpoint flanked by KMT2A (chromosome 11, exon 10) and AFDN (Chromosome 6, exon 2). C) Chromatogram of Sanger DNA-seq showing the observed breakpoint in the cDNA reverse transcribed from patient 7’s RNA isolated from the bone marrow sample at relapse. D) A Chromatogram of Sanger DNA-seq shows the observed breakpoint in the cDNA reverse transcribed from patient 20’s RNA isolated from the refractory bone marrow sample (after treatment). Please note Chromosome 11-exon 9 (indicated by a blue circle) flanks the breakpoint of KMT2A/AFDN after treatment.

Although both patients 7 and 20 carried the KMT2A/AFDN gene fusion, we aimed to determine if they shared similar breakpoints. Patient 7’s KMT2A/AFDN gene fusion exhibited breakpoints in KMT2A exon 9 and AFDN exon 2 (KMT2A exon 9::AFDN exon 2). To analyze the breakpoints in Patient 20, PCR was performed on both cDNA and gDNA, followed by Sanger DNA sequencing of the bone marrow sample obtained at diagnosis. The primers used are listed in Supplementary Table 1. The analysis revealed that Patient 20 had a chimeric chromosome with a breakpoint flanked by KMT2A exon 10 and AFDN exon 2, indicating differing variants of the same KMT2A/AFDN gene fusion between patients 7 and 20 (Fig. 8A and B).

### KMT2A/AFDN gene fusion variants and treatment-driven clonal selection

Following the identification of differing variants of the KMT2A/AFDN gene fusion between patients 7 and 20, we delved into the treatment-driven clonal selection underlying these variations. However, the unexpected correlation between a significantly reduced chimeric KMT2A/AFDN gene expression and the refractory disease in Patient 20 prompted us to investigate the possibility of unknown fusion genes emerging post-treatment, potentially contributing to the unfavorable clinical outcome. Despite thorough PCR and Sanger DNA sequencing analyses, we did not identify the presence of the unknown CCDC32/CBX3 fusion gene in the specimens collected at the diagnosis and relapse of Patient 20.

We revisited the breakpoints analysis of both Patient 7 and Patient 20 to gain further insight into the mechanisms behind refractory/relapse in KMT2A/AFDN rearrangements and determine if the same gene fusion persisted in both patients during treatment. Patient 7 exhibited no significant change in the KMT2A/AFDN fusion gene breakpoints at diagnosis and during relapse (Fig. 8A and C). Conversely, in Patient 20, we identified a different breakpoint in the KMT2A gene in the relapsed bone marrow sample post-treatment. Initially, Patient 20’s breakpoint was flanked by KMT2A exon 10 and AFDN exon 2 (Fig. 8B), but after treatment, it shifted to KMT2A exon 9 and AFDN exon 2 (Fig. 8D), mirroring Patient 7’s breakpoint (KMT2A exon 9::AFDN exon 2, Fig. 8C).

**Fig. 9.**
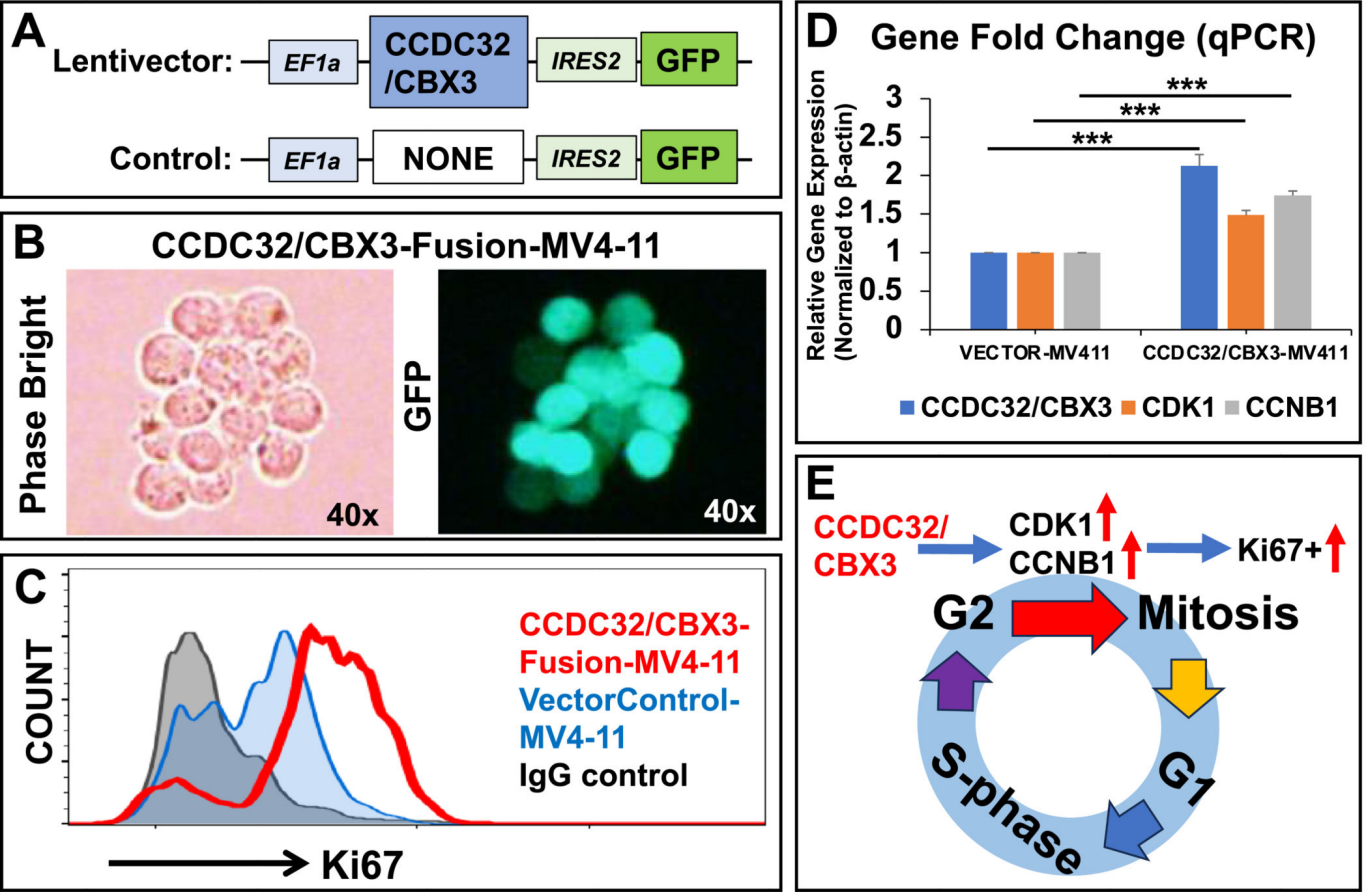
Functional validation of the CCDC32/CBX fusion gene demonstrates its promotion of cell cycle progression in AML blasts in vitro, as revealed by ectopic overexpression in a new transgenic cell line - patient-specific MV4–11 Cell Line (FLT3-Mutated AML).A) Schematic diagram of a lentiviral expression construct containing CCDC32/CBX3 fusion gene and eGFP reporter, with promoters EF1a and IRES2, respectively; The custom-built lentiviral construct was obtained from GeneCopoeia with catalog #: CS-GS2239L-Lv225–01. B) The images (phase bright and fluorescent) of the same leukemia cluster reveal that most CCDC32/CBX3-MV4–11 cells are GFP+ ; C) Representative flow cytometry histogram shows the increased Ki67 + expressions of CCDC32/CBX3-MV4–11 cells;D) Gene expressions of CCDC32/CBX3, CDK1 and CCNB1 in CCDC32/CBX3-MV4–11 cells, analyzed by qPCR. Data of mRNA expressions show the fold change (normalized to β-actin) of different genes in CCDC32/CBX3-MV4–11 cells versus vector-control cells. Where applicable, data are means ± SEM. * **P < 0.005, N = 3. E) Potential Impact of the CCDC32/CBX Fusion Protein on Cell Cycle Progression in the MV4–11 Cell Line (FLT3-Mutated AML) *In Vitro.*

These findings indicated that Patient 20 harbored multiple subclones, with a dominant clone featuring the KMT2A exon 10::AFDN exon 2 gene fusion, and a minor clone featuring the KMT2A exon 9::AFDN exon 2 gene fusion. Notably, the clone with the KMT2A exon 10::AFDN exon 2 fusion was sensitive to treatment, as evidenced by the drastic 103-fold decrease in the expression of the fusion KMT2A/AFDN mRNA (Fig. 7E). It is plausible that the subclone with the distinct breakpoint flanked by KMT2A exon 9 and AFDN exon 2 may be accountable for the refractory disease observed in Patient 20. We further wanted the functional validation with experimental evidence demonstrating the biological impact of the CCDC32/CBX3 fusion on AML pathogenesis or treatment resistance using patient-specific MV4–11 Cell Line (FLT3-Mutated AML) (Fig. 9). We created a new transgenic cell line of ectopic overexpression of CCDC32/CBX fusion gene using a lentiviral expression construct containing CCDC32/CBX3 fusion gene and eGFP reporter, with promoters EF1a and IRES2 (Fig. 9A). A flow cytometry histogram shows that the expression of CCDC32/CBX3-MV4–11 increased the number of Ki67 + cells compared to vector controls (Fig. 9C). Gene expressions of CCDC32/CBX3, CDK1 and CCNB1 in CCDC32/CBX3-MV4–11 cells increased as analyzed by qPCR (Fig. 9D). Thus, CCDC32/CBX fusion protein promoted the cell cycle progression of MV4–11 (an *FLT3*-mutated AML cell line) *in vitro* (Fig. 9E).

## Discussion

Among 16 patients in this study, while patients 7 and 20 initially presented with the KMT2A/AFDN gene fusion, patient 20 exhibited the absence of this fusion at relapse, indicating a subclonal evolution possibly driven by drug treatment (Fig. 1A). Conversely, patient 7 maintained the KMT2A/AFDN fusion both at diagnosis and relapse. Notably, patient 7 retained the CCDC32/CBX3 gene fusion throughout their diagnostic and relapse stages (Fig. 5). Both patients 7 and 20 had a similar diagnosis of AML with a t(6;11)(q27;q23) translocation leading to a KMT2A/AFDN gene fusion (Fig. 8). Patient 7 underwent relapse 2 years after remission before passing away, while patient 20 expired a year and a half after initial treatment without undergoing her presumed stem cell transplantation (Fig. 1B). These data might provide the differential mechanisms of refractory/relapsed adult AML patients.

Distinctly, in patient 7, we found a cytogenetically unknown CCDC32/CBX3 gene fusion that had been previously undetected by standard cytogenetic tools like FISH and chromosome analysis (Figs. 3–5). Thus, we confirmed the necessity of RNA-seq and molecular profiling to detect gene fusions that may be missed by conventional cytogenetic tools, which align with previous publications (Heyer et al., 2019; Kerbs et al., 2022; Li et al., 2023). Furthermore, we identified another 3 patients with relapsed AML possessing this unknown CCDC32/CBX3 gene fusion, suggesting it’s not an exclusive event for patient 7; however, our study does not have sufficient data to demonstrate any clinical or experimental value of the AML-related CCDC32/CBX3 fusion, even though this fusion gene impact has been reported in MYCN non-amplified neuroblastoma related dual fusion genes (NB) (Lee et al., 2020).

In 58 whole exome sequencing (WES) and 48 whole transcriptome sequencing (WTS) samples of MYCN non-amplified neuroblastoma (NB), recurrent mutations were observed in genes like MUC4 and RBMXL3. In contrast, dual gene fusions (CCDC32-CBX3 [10 %]and SAMD5-SASH1 [6 %]) were prevalent, especially in non-high-risk patients with ganglioneuroblastoma histology(Lee et al., 2020). Their risk-group-specific biomarkers showed differential gene expression, with immune-related pathways activated in the high-risk group. Mutational signatures and tumor mutation burden (TMB) analyses of those patient profiles revealed potential targets for immunotherapeutic strategies in NB. In vitro knockdown of the chimeric CCDC32/CBX3 mRNA led to reduced cell proliferation and motility in astrocytes and a prostate cell line (Singh et al., 2020). Additionally, CCDC32/CBX3 gene fusion has also been detected in primary tumors and metastatic lesions from papillary thyroid carcinoma patients (Stenman et al., 2021).

Separately, studies have shown CBX3 is an oncogene for many cancers (Peng et al., 2022; Zhang et al., 2022), but the function of CCDC32 in cancers remains to be elucidated. A previous study reported that prostate cancer-associated transcript 18 (PCAT-18), a long non-coding RNA, interacted with CCDC32 as a tumor suppressor in gastric cancers (Foroughi et al., 2018). Additionally, the CCDC family of proteins, including CCDC67 or CCDC6, are known to play functional roles as tumor suppressors (Morra et al., 2021; Yin et al., 2016). Interestingly, we found that the expression of the wild-type CBX3 gene (probable oncogene) significantly increased during relapse. In contrast, wild-type CCDC32 (possible tumor suppressor) decreased, with the CCDC32/CBX3 gene fusion unchanged (Fig. 6B). These observations raise the intriguing possibility that the chimera plays a role in modulating wild-type CCDC32 and CBX3 gene expression to promote relapse without requiring its own expression to be changed. If so, there is a need to investigate both physiological consequences of affected normal genes in relapsed/refractory AML and functional roles of the lesser known, cytogenetically unknown gene fusions like CCDC32/CBX3 so that targeted therapies can be more specific and effective.

Complex genomic rearrangements, which are structural variants with more than one breakpoint, have been under-studied and underrecognized for their role in causing diseases (Issa et al., 2021; Maroilley et al., 2023). Originally, newly diagnosed patients 7 and 20 harbored differing breakpoints in their DNAs (Fig. 8); however, after treatment, patient 20 harbored the same breakpoint of KMT2A exon 9:: AFDN exon 2 as patient 7 (Fig. 8D). This changing of breakpoints suggests the presence of a complex genomic rearrangement in the KMT2A:: AFDN oncofusion gene. Our result is consistent with a previous study showing multiple breakpoints (though more often with breakpoints in exon/intron 9 of KMT2A) in AML patients with KMT2A/AFDN translocations (Meyer et al., 2018). MACE-Seq (Massive Analysis of cDNA Ends Sequencing), used to analyze molecular functions of different KMT2A/AFDN fusion proteins in transgenic cell lines, revealed that KMT2A exon 9::AFDN exon 2 fusion protein activated more de novo genes (4748 genes) than KMT2A exon 10::AFDN exon 2 (2551 genes) did (Kundu et al., 2021). Our results of patient 7 showing that the KMT2A exon 9::AFDN exon 2 gene fusion drastically increased in the relapsed sample of patient 7 compared to the initial diagnosis (Fig. 6a) supported the clonal selection of the exon 9 fusion protein, potentially leading to a more aggressive variant than the exon 10 variant. KMT2A rearrangements are known to have an unfavorable prognosis in AML, and the specific t(6;11)(q27;23) translocation in AML patients leads to a median overall survival of only 12 months (Berg et al., 2022; Schuy et al., 2022). Our novel findings (Fig. 8) also suggest that one reason for the adverse outcomes of KMT2A chromosomal rearrangements is their complex ability to generate chimeric variants and more aggressive subclones to elude the treatment, thereby subclonal switching mechanisms from dominating to dormant phases(Li et al., 2012).

Overall, our study implies that standard cytogenetic analyses and emerging NGS (Haas et al., 2019) are insufficient to diagnose a patient with chromosome rearrangement fully. Mainly, short-read RNA-seq presents problems because it cannot detect gene fusions with low expression levels (Heyer et al., 2019), like the KMT2A/AFDN gene fusion that was undetected in patient 7 by our short-read RNA-seq (Fig. 3). Instead, long-read RNA-seq, combined with cytogenetics and molecular profiling through PCR/Sanger DNA-sequencing, should be utilized to identify complex fusion transcripts and different chimeric variants of multiple gene fusions (Davidson et al., 2022; Mariya et al., 2023). When we utilized RNA-seq for patients 7 and 20, we identified CCDC32/CBX3 from patient 7 and confirmed by PCR/Sanger DNA-sequencing with primers specifically designed for each candidate of gene fusions. Thus, we envision long-read RNA-seq being used as an initial screening tool to identify all potential candidates of gene fusions, combined with multilayer confirmatory tools (Fig. 1A), including chromosome analysis/FISH and PCR/Sanger DNA-sequencing to diagnose the whole landscape of chromosome rearrangements. A similar strategy of combining whole-genome Sequencing (WGS) and confirmatory approaches has been found to provide more significant diagnostic profiling and better accuracy than routine cytogenetic analyses in patients with AML or myelodysplastic syndromes (MDS) at the genomic level (Duncavage et al., 2021), except for the apparent weakness without providing transcriptome resolution. Currently, the frequency of unknown AML gene fusions is unknown (Stengel et al., 2020). Still, our proposed diagnostic plan (Fig. 1A) could be essential for identifying undetected gene fusions and their mechanisms of action in refractory/relapsed AML, leading to finding an eventual cure for cancer patients with chromosome rearrangement.

We found the fusion gene (CCDC32/CBX3) in patient 7, which has not been reported in AML, and which is novel in AML. However, the fusion gene (CCDC32/CBX3) is not “The hypothesized gene fusion” but has been independently identified by two laboratories in 2020. The first is titled “Genomic profile of MYCN non-amplified neuroblastoma and potential for immunotherapeutic strategies in neuroblastoma” (Lee et al., 2020), showing that the fusion gene of CCDC32-CBX3 exists in 10 % of neuroblastoma patients. The second, entitled “The landscape of chimeric RNAs in non-diseased tissues and cells”(Singh et al., 2020), illustrates their novel technology of The landscape of chimeric RNAs to identify the fusion genes, a technology that was developed by Two well-known oncogene expert hunters, Hui Li and his collaborator, Jeffrey Sklar. Specifically, our manuscript determines that the fusion gene (CCDC32/CBX3) is present in AML, using the same technology for searching for the fusion gene (Singh et al., 2020). Singh and colleagues 1) clearly marked the fusion breakpoint (the same fusion gene we reported); 2) did a knock-out of function, showing the existence of a protein. We did the function validation in Fig. 9 - using overexpression of CCDC32/CBX3 fusion gene in MV4–11 AML cells for the functional validation with experimental evidence to demonstrate the biological impact of the CCDC32/CBX3 fusion on AML pathogenesis or treatment resistance mechanism by which CCDC32/CBX fusion protein promotes the cell cycle progression with patient-specific MV4–11 Cell Line (FLT3-Mutated AML) (Fig. 9); 3) they did not do patients but We did it to show novelty in AML patients.

We want to address the limitations of the study to provide a comprehensive framework for future research to guide further investigations aimed at addressing these gaps. One significant limitation is the sample size, as our findings are based on two patients with the KMT2A/AFDN translocation, which restricts the generalizability of the results and underscores the need for larger cohort studies involving AML patients with t(6;11)(q27;q23). Additionally, the study focuses predominantly on genetic alterations, leaving protein-level expression and the functional consequences of the chimeric fusions unexplored, which are critical for understanding the full biological and clinical implications. Another limitation stems from the reliance on short-read RNA sequencing for the initial analysis, which may have missed low-abundance fusion transcripts, highlighting the need for long-read RNA sequencing and confirmatory methods in future studies. Therefore, future studies could investigate the protein expression of the chimeras and how they may change after treatment or relapse. To examine the function of lesser-known gene fusions like CCDC32/CBX3 in AML, future gain or loss of function studies could also be executed to characterize subclonal evolution evolving treatment (Lee and Li, 2020).

To underscore the clinical implications of our findings for diagnosis, therapy, and prognosis in AML, we propose a multilayer diagnostic strategy combining long-read RNA sequencing, FISH, and PCR/Sanger DNA sequencing to detect complex gene fusions and subclonal evolution. This approach aligns with emerging precision oncology strategies for refractory or relapsed AML cases. Furthermore, the study highlights the importance of investigating the functional roles of underexplored fusions, such as CCDC32/CBX3, in AML progression, as these may reveal therapeutic vulnerabilities, including targeting wild-type CBX3 overexpression during relapse. From a prognostic standpoint, the variability in gene fusion patterns and breakpoints, particularly in KMT2A/AFDN, provides valuable insights into relapse risks and disease aggressiveness, reinforcing the need for personalized treatment strategies. Lastly, the study’s relevance is further enhanced by its contributions to understanding clonal selection mechanisms and its broader implications in cancer biology, as demonstrated by parallels with other malignancies, including neuroblastoma and thyroid carcinoma. These additions offer a more comprehensive view of the translational potential of our findings.

## Conclussion

In conclusion, our study provides critical insights into the clinical implications of complex gene fusions and subclonal evolution in AML, offering a multilayer diagnostic strategy, highlighting therapeutic vulnerabilities in underexplored fusions like CCDC32/CBX3, emphasizing the prognostic significance of fusion variability in relapse risk, and demonstrating broader relevance through parallels with other malignancies, thus paving the way for personalized treatment strategies and advancing cancer biology of drug resistance.

## Supplementary Material

Supplemental Table

## Figures and Tables

**Table 1:** Clinical profiles of seven newly diagnosed AML patients with chromosomal abnormalities and their corresponding clinical outcomes. (Note that the novel AML fusion gene was detected n = 2 out of 16 patients screened).

	Age	Sex	WBC	Cytogenetic Findings	Clinical Outcome

Patient 2	72	F	82 K w/ 87 % blasts	47,XX,inv(3)(q21q26.2),+ 13[20]	alive
Patient 6	75	F	26, w/ 80–90 % blast	46,XX,del(4)(q31)[2]/46,XX[18]	alive
Patient 7	21	M	42 K, 76 % blast	46,XY,t(6,11)(q27;q23)[11]/46,XY[9] KMT2A (MLL) 11q23 rearrangement	deceased
Patient 15	28	F	47 K, 49 % blast	inv(16)(p13.1q22); CBFB/MYH11	alive
Patient 18	56	M	74.7 K, 26 % blast	inv(16), or t(16,16); MYH11/CBFB	deceased
Patient 20	35	F	17.8 K, 80 % blast	46,XX,del(6)(q13q27),der(11)t(6;11)(q27;q23)dup(11)(q21q23)t(6;11)[5]/54,idem,+ 6,+ 7,+ 8,+ 19,+ 21,+ 22 [15]complex 6;11 translocation results in AFDN/KMT2A gene fusion	deceased
Patient 22	58	M	14.3 K, 9 % blast	t(8;21); RUNX1T1/RUNX1 gene fusion	alive

## Data Availability

Transcriptomic data for RNA-seq and Sanger DNA-seq data deposited at the Gene Expression Omnibus (GEO) are available for all investigators. The sequences of the primers are available in Supplementary Table 1. The raw data sets were deposited onto the database of the NCBI (The National Center for Biotechnology Information) of the NIH (the National Institutes of Health) with the bioproject number PRJNA1201248.

## References

[R1] Arindrarto W, Borras DM, de Groen RAL, van den Berg RR, Locher IJ, van Diessen S, van der Holst R, van der Meijden ED, Honders MW, de Leeuw RH, Verlaat W, Jedema I, Kroes WGM, Knijnenburg J, van Wezel T, Vermaat JSP, Valk PJM, Janssen B, de Knijff P, van Bergen CAM, van den Akker EB, Hoen PAC, Kielbasa SM, Laros JFJ, Griffioen M, Veelken H, 2021. Comprehensive diagnostics of acute myeloid leukemia by whole transcriptome RNA sequencing. Leukemia 35, 47–61. 32127641 10.1038/s41375-020-0762-8 PMC7787979

[R2] Berg HE, Blackburn PR, Smadbeck JB, Swanson KE, Rice CS, Webley MR, Johnson SH, Vasmatzis G, Xu X, Greipp PT, Hoppman NL, Ketterling RP, Baughn LB, Boston CH, Sutton LM, Peterson JF, 2021. Detection of a Cryptic EP300/ZNF384 gene fusion by chromosomal microarray and next-generation sequencing studies in a pediatric patient with b-lymphoblastic leukemia. Lab Med 52, 297–302. 33145596 10.1093/labmed/lmaa085

[R3] Berg HE, Greipp PT, Baughn LB, Falcon CP, Jackson CC, Peterson JF, 2022. Detection of a Cryptic KMT2A/AFDN Gene Fusion [ins(6;11)(q27;q23q23)] in a pediatric patient with newly diagnosed acute myeloid leukemia. Lab Med 53, e95–e99. 34894139 10.1093/labmed/lmab109

[R4] Bill M, Mrozek K, Kohlschmidt J, Eisfeld AK, Walker CJ, Nicolet D, Papaioannou D, Blachly JS, Orwick S, Carroll AJ, Kolitz JE, Powell BL, Stone RM, de la Chapelle A, Byrd JC, Bloomfield CD, 2020. Mutational landscape and clinical outcome of patients with de novo acute myeloid leukemia and rearrangements involving 11q23/KMT2A. Proc. Natl. Acad. Sci. USA 117, 26340–26346. 33020282 10.1073/pnas.2014732117 PMC7584992

[R5] Cao H, Xiao J, Reeves ME, Payne K, Chen CS, Baylink DJ, Marcucci G, Xu Y, 2020. Discovery of proangiogenic CD44+mesenchymal cancer stem cells in an acute myeloid leukemia patient’s bone marrow. J. Hematol. Oncol 13, 63. 32493379 10.1186/s13045-020-00899-x PMC7268388

[R6] Davidson NM, Chen Y, Sadras T, Ryland GL, Blombery P, Ekert PG, Goke J, Oshlack A, 2022. JAFFAL: detecting fusion genes with long-read transcriptome sequencing. Genome Biol. 23, 10. 34991664 10.1186/s13059-021-02588-5 PMC8739696

[R7] Dohner H, Weisdorf DJ, Bloomfield CD, 2015. Acute Myeloid Leukemia. N. Engl. J. Med 373, 1136–1152. 26376137 10.1056/NEJMra1406184

[R8] Duncavage EJ, Schroeder MC, O’Laughlin M, Wilson R, MacMillan S, Bohannon A, Kruchowski S, Garza J, Du F, Hughes AEO, Robinson J, Hughes E, Heath SE, Baty JD, Neidich J, Christopher MJ, Jacoby MA, Uy GL, Fulton RS, Miller CA, Payton JE, Link DC, Walter MJ, Westervelt P, DiPersio JF, Ley TJ, Spencer DH, 2021. Genome sequencing as an alternative to cytogenetic analysis in myeloid cancers. N. Engl. J. Med 384, 924–935. 33704937 10.1056/NEJMoa2024534 PMC8130455

[R9] Foroughi K, Amini M, Atashi A, Mahmoodzadeh H, Hamann U, Manoochehri M, 2018. Tissue-specific down-regulation of the long non-coding RNAs PCAT18 and LINC01133 in gastric cancer development. Int J. Mol. Sci 19. 10.3390/ijms19123881 PMC6321575 30518158

[R10] Haas BJ, Dobin A, Li B, Stransky N, Pochet N, Regev A, 2019. Accuracy assessment of fusion transcript detection via read-mapping and de novo fusion transcript assembly-based methods. Genome Biol. 20, 213. 31639029 10.1186/s13059-019-1842-9 PMC6802306

[R11] Heyer EE, Deveson IW, Wooi D, Selinger CI, Lyons RJ, Hayes VM, O’Toole SA, Ballinger ML, Gill D, Thomas DM, Mercer TR, Blackburn J, 2019. Diagnosis of fusion genes using targeted RNA sequencing. Nat. Commun 10, 1388. 30918253 10.1038/s41467-019-09374-9 PMC6437215

[R12] Hoffmeister LM, Orhan E, Walter C, Niktoreh N, Hanenberg H, von Neuhoff N, Reinhardt D, Schneider M, 2021. Impact of KMT2A Rearrangement and CSPG4 expression in pediatric acute myeloid leukemia. Cancers (Basel) 13. 10.3390/cancers13194817 PMC8508499 34638301

[R13] Issa GC, Zarka J, Sasaki K, Qiao W, Pak D, Ning J, Short NJ, Haddad F, Tang Z, Patel KP, Cuglievan B, Daver N, DiNardo CD, Jabbour E, Kadia T, Borthakur G, Garcia-Manero G, Konopleva M, Andreeff M, Kantarjian HM, Ravandi F, 2021. Predictors of outcomes in adults with acute myeloid leukemia and KMT2A rearrangements. Blood Cancer J. 11, 162. 34588432 10.1038/s41408-021-00557-6 PMC8481264

[R14] Kantarjian H, 2016. Acute myeloid leukemia–major progress over four decades and glimpses into the future. Am. J. Hematol 91, 131–145. 26598393 10.1002/ajh.24246

[R15] Kerbs P, Vosberg S, Krebs S, Graf A, Blum H, Swoboda A, Batcha AMN, Mansmann U, Metzler D, Heckman CA, Herold T, Greif PA, 2022. Fusion gene detection by RNA-sequencing complements diagnostics of acute myeloid leukemia and identifies recurring NRIP1-MIR99AHG rearrangements. Haematologica 107, 100–111. 34134471 10.3324/haematol.2021.278436 PMC8719081

[R16] Kumar CC, 2011. Genetic abnormalities and challenges in the treatment of acute myeloid leukemia. Genes Cancer 2, 95–107. 21779483 10.1177/1947601911408076 PMC3111245

[R17] Kundu A, Kowarz E, Marschalek R, 2021. The role of reciprocal fusions in MLL-r acute leukemia: studying the chromosomal translocation t(6;11). Oncogene 40, 5902–5912. 34354240 10.1038/s41388-021-01983-3 PMC8497272

[R18] Lee E, Lee JW, Lee B, Park K, Shim J, Yoo KH, Koo HH, Sung KW, Park WY, 2020. Genomic profile of MYCN non-amplified neuroblastoma and potential for immunotherapeutic strategies in neuroblastoma. BMC Med Genom. 13, 171. 10.1186/s12920-020-00819-5 PMC7653769 33172452

[R19] Lee LX, Li SC, 2020. Hunting down the dominating subclone of cancer stem cells as a potential new therapeutic target in multiple myeloma: An artificial intelligence perspective. World J. Stem Cells 12, 706–720, 2020 August; doi: 20 10.4252/wjsc.v2012.i2028.2706. 32952853 PMC7477658

[R20] Li SC, Lee KL, Luo J, 2012. Control dominating subclones for managing cancer progression and posttreatment recurrence by subclonal switchboard signal: implication for new therapies. Stem Cells Dev. 21, 503–506. 21933025 10.1089/scd.2011.0267 PMC6916525

[R21] Li SC, Tachiki LM, Kabeer MH, Dethlefs BA, Anthony MJ, Loudon WG, 2014. Cancer genomic research at the crossroads: realizing the changing genetic landscape as intratumoral spatial and temporal heterogeneity becomes a confounding factor. Cancer Cell Int. 14, 115 (pages 111–116). 25411563 10.1186/s12935-014-0115-7 PMC4236490

[R22] Li Y, Liu Y, Gao X, Zhao W, Zhou F, Liu H, Wang W, 2023. Identification of novel PIEZO1::CBFA2T3 and INO80C::SETBP1 fusion genes in an acute myeloid leukemia patient by RNA-seq. Mol. Biol. Rep 50, 1961–1966. 36472727 10.1007/s11033-022-08138-x

[R23] Mariya T, Shichiri Y, Sugimoto T, Kawamura R, Miyai S, Inagaki H, Sugihara E, Ikeda K, Baba T, Ishikawa A, Ammae M, Nakaoka Y, Saito T, Sakurai A, Kurahashi H, 2023. Clinical application of long-read nanopore sequencing in a preimplantation genetic testing pre-clinical workup to identify the junction for complex Xq chromosome rearrangement-related disease. Prenat. Diagn 43, 304–313. 36797813 10.1002/pd.6334

[R24] Maroilley T, Flibotte S, Jean F, Rodrigues Alves Barbosa V, Galbraith A, Chida AR, Cotra F, Li X, Oncea L, Edgley M, Moerman D, Tarailo-Graovac M, 2023. Genome sequencing of C. elegans balancer strains reveals previously unappreciated complex genomic rearrangements. Genome Res 33, 154–167. 36617680 10.1101/gr.276988.122 PMC9977149

[R25] Meyer C, Burmeister T, Groger D, Tsaur G, Fechina L, Renneville A, Sutton R, Venn NC, Emerenciano M, Pombo-de-Oliveira MS, Barbieri Blunck C, Almeida Lopes B, Zuna J, Trka J, Ballerini P, Lapillonne H, De Braekeleer M, Cazzaniga G, Corral Abascal L, van der Velden VHJ, Delabesse E, Park TS, Oh SH, Silva MLM, Lund-Aho T, Juvonen V, Moore AS, Heidenreich O, Vormoor J, Zerkalenkova E, Olshanskaya Y, Bueno C, Menendez P, Teigler- Schlegel A, Zur Stadt U, Lentes J, Gohring G, Kustanovich A, Aleinikova O, Schafer BW, Kubetzko S, Madsen HO, Gruhn B, Duarte X, Gameiro P, Lippert E, Bidet A, Cayuela JM, Clappier E, Alonso CN, Zwaan CM, van den Heuvel-Eibrink MM, Izraeli S, Trakhtenbrot L, Archer P, Hancock J, Moricke A, Alten J, Schrappe M, Stanulla M, Strehl S, Attarbaschi A, Dworzak M, Haas OA, Panzer-Grumayer R, Sedek L, Szczepanski T, Caye A, Suarez L, Cave H, Marschalek R, 2018. The MLL recombinome of acute leukemias in 2017. Leukemia 32, 273–284. 28701730 10.1038/leu.2017.213 PMC5808070

[R26] Morra F, Merolla F, Zito Marino F, Catalano R, Franco R, Chieffi P, Celetti A, 2021. The tumour suppressor CCDC6 is involved in ROS tolerance and neoplastic transformation by evading ferroptosis. Heliyon 7, e08399. 34841108 10.1016/j.heliyon.2021.e08399 PMC8605351

[R27] Peng W, Shi S, Zhong J, Liang H, Hou J, Hu X, Wang F, Zhang J, Geng S, Sun X, Zhong D, Cui H, 2022. CBX3 accelerates the malignant progression of glioblastoma multiforme by stabilizing EGFR expression. Oncogene 41, 3051–3063. 35459780 10.1038/s41388-022-02296-9

[R28] Schuy J, Grochowski CM, Carvalho CMB, Lindstrand A, 2022. Complex genomic rearrangements: an underestimated cause of rare diseases. Trends Genet 38, 1134–1146. 35820967 10.1016/j.tig.2022.06.003 PMC9851044

[R29] Singh S, Qin F, Kumar S, Elfman J, Lin E, Pham LP, Yang A, Li H, 2020. The landscape of chimeric RNAs in non-diseased tissues and cells. Nucleic Acids Res 48, 1764–1778. 31965184 10.1093/nar/gkz1223 PMC7038929

[R30] Stengel A, Shahswar R, Haferlach T, Walter W, Hutter S, Meggendorfer M, Kern W, Haferlach C, 2020. Whole transcriptome sequencing detects a large number of novel fusion transcripts in patients with AML and MDS. Blood Adv. 4, 5393–5401. 33147338 10.1182/bloodadvances.2020003007 PMC7656918

[R31] Stenman A, Backman S, Johansson K, Paulsson JO, Stalberg P, Zedenius J, Juhlin CC, 2021. Pan-genomic characterization of high-risk pediatric papillary thyroid carcinoma. Endocr. Relat. Cancer 28, 337–351. 33827048 10.1530/ERC-20-0464 PMC8111328

[R32] Thol F, 2022. Fusion genes in acute myeloid leukemia: do acute myeloid leukemia diagnostics need to fuse with RNA-sequencing? Haematologica 107, 44–45. 34134473 10.3324/haematol.2021.278983 PMC8719096

[R33] Untergasser A, Cutcutache I, Koressaar T, Ye J, Faircloth BC, Remm M, Rozen SG, 2012. Primer3–new capabilities and interfaces. Nucleic Acids Res 40, e115. 22730293 10.1093/nar/gks596 PMC3424584

[R34] Wheeler FC, Kim AS, Mosse CA, Shaver AC, Yenamandra A, Seegmiller AC, 2018. Limited utility of fluorescence in situ hybridization for recurrent abnormalities in acute myeloid leukemia at diagnosis and follow-up. Am. J. Clin. Pathol 149, 418–424. 10.1093/ajcp/aqy002 PMC5888921 29538617

[R35] Winters AC, Bernt KM, 2017. MLL-Rearranged leukemias-an update on science and clinical approaches. Front Pedia 5, 4. 10.3389/fped.2017.00004 PMC5299633 28232907

[R36] Xu Y, Hino C, Baylink DJ, Xiao J, Reeves ME, Zhong JF, Mirshahidi S, Cao H, 2022a. Vitamin D activates FBP1 to block the Warburg effect and modulate blast metabolism in acute myeloid leukemia. Biomark. Res 10, 16. 35366947 10.1186/s40364-022-00367-3 PMC8977002

[R37] Xu Y, Lopes C, Qian Y, Liu Y, Cheng L, Goulding M, Turner EE, Lima D, Ma Q, 2008. Tlx1 and Tlx3 coordinate specification of dorsal horn pain-modulatory peptidergic neurons. J. Neurosci 28, 4037–4046. 18400903 10.1523/JNEUROSCI.4126-07.2008 PMC2681187

[R38] Xu Y, Tran L, Tang J, Nguyen V, Sewell E, Xiao J, Hino C, Wasnik S, Francis-Boyle OL, Zhang KK, Xie L, Zhong JF, Baylink DJ, Chen CS, Reeves ME, Cao H, 2022b. FBP1-Altered carbohydrate metabolism reduces leukemic viability through activating P53 and modulating the mitochondrial quality control system in vitro. Int J. Mol. Sci 23. 10.3390/ijms231911387 PMC9570078 36232688

[R39] Yin DT, Xu J, Lei M, Li H, Wang Y, Liu Z, Zhou Y, Xing M, 2016. Characterization of the novel tumor-suppressor gene CCDC67 in papillary thyroid carcinoma. Oncotarget 7, 5830–5841. 26716505 10.18632/oncotarget.6709 PMC4868724

[R40] Zhang P, Yang X, Zha Z, Zhu Y, Zhang G, Li G, 2022. CBX3 regulated by miR-139 promotes the development of HCC by regulating cell cycle progression. Cell Cycle 21, 1740–1752. 35471148 10.1080/15384101.2022.2068329 PMC9302499

