# Supplementary material for: Exploring treatment-driven subclonal evolution of prognostic triple biomarkers: Dual gene fusions and chimeric RNA variants in novel subtypes of acute myeloid leukemia patients with KMT2A rearrangement
Source: Drug Resist Updat. Author manuscript; Available in PMC 2025 May 13. (PMC12069159; doi:10.1016/j.drup.2024.101199)
Supplement: Supplemental Table [file NIHMS2069981-supplement-Supplemental_Table.pdf]

Supplementary Table 1: List of Primers (* OriGene) used in this study

#	Name (HUMAN)	Forward Sequence	Reverse Sequence
1	**KMT2A***	GTGCTTTGTGGTCAGCGGAAGT	TGTGAGACAGCAACCCACGGTG
2	**AFDN ***	AGTCGGTTGTGAAAGGAGGTGC	TCCTGAGAGAGTCCAACCAGAC
3	**KMT2A/AFDN**	TACCCCATCAGCAAGAGAGG	CTTGGGAGAGGACAGCATTC
4	**CCDC32 ***	CGGTTCCTCCAGGAGAAGTTAG	CCTGTGACTCTGGAGGAATCAG
5	**CBX3 ***	GCTGACAAACCAAGAGGATTTGC	CAGCACCAAGTCTGCCTCATCT
6	**CCDC32/CBX3**	GAAGATGGTGCCAACAATGC	GCTATTATTCACCGCCTCCG
